# A dual role of lysophosphatidic acid type 2 receptor (LPAR2) in nonsteroidal anti-inflammatory drug-induced mouse enteropathy

**DOI:** 10.1038/s41401-023-01175-7

**Published:** 2023-10-10

**Authors:** Barbara Hutka, Anett Várallyay, Szilvia B. László, András S. Tóth, Bálint Scheich, Sándor Paku, Imre Vörös, Zoltán Pós, Zoltán V. Varga, Derek D. Norman, Andrea Balogh, Zoltán Benyó, Gábor Tigyi, Klára Gyires, Zoltán S. Zádori

**Affiliations:** 1 Department of Pharmacology and Pharmacotherapy, Semmelweis University, Budapest, Hungary; 2 Pharmacological and Drug Safety Research, Gedeon Richter Plc, Budapest, Hungary; 3 Department of Pathology and Experimental Cancer Research, Semmelweis University, Budapest, Hungary; 4 HCEMM-SU Cardiometabolic Immunology Research Group, Semmelweis University, Budapest, Hungary; 5 MTA-SE Momentum Cardio-Oncology and Cardioimmunology Research Group, Budapest, Hungary; 6 MTA-SE System Pharmacology Research Group, Budapest, Hungary; 7 Department of Genetics, Cell and Immunobiology, Semmelweis University, Budapest, Hungary; 8 Department of Physiology, College of Medicine, University of Tennessee Health Science Center (UTHSC), Memphis, TN USA; 9 Institute of Translational Medicine, Semmelweis University, Budapest, Hungary; 10 HUN-REN–SU Cerebrovascular and Neurocognitive Diseases Research Group, Budapest, Hungary

**Keywords:** enteropathy, nonsteroidal anti-inflammatory drug, lysophosphatidic acid, LPAR2, DBIBB, autotaxin

## Abstract

Lysophosphatidic acid (LPA) is a bioactive phospholipid mediator that has been found to ameliorate nonsteroidal anti-inflammatory drug (NSAID)-induced gastric injury by acting on lysophosphatidic acid type 2 receptor (LPAR2). In this study, we investigated whether LPAR2 signaling was implicated in the development of NSAID-induced small intestinal injury (enteropathy), another major complication of NSAID use. Wild-type (WT) and *Lpar2* deficient (*Lpar2*^*−/−*^) mice were treated with a single, large dose (20 or 30 mg/kg, i.g.) of indomethacin (IND). The mice were euthanized at 6 or 24 h after IND treatment. We showed that IND-induced mucosal enteropathy and neutrophil recruitment occurred much earlier (at 6 h after IND treatment) in *Lpar2*^*−/−*^ mice compared to WT mice, but the tissue levels of inflammatory mediators (IL-1β, TNF-α, inducible COX-2, CAMP) remained at much lower levels. Administration of a selective LPAR2 agonist DBIBB (1, 10 mg/kg, i.g., twice at 24 h and 30 min before IND treatment) dose-dependently reduced mucosal injury and neutrophil activation in enteropathy, but it also enhanced IND-induced elevation of several proinflammatory chemokines and cytokines. By assessing caspase-3 activation, we found significantly increased intestinal apoptosis in IND-treated *Lpar2*^*−/−*^ mice, but it was attenuated after DBIBB administration, especially in non-obese diabetic/severe combined immunodeficiency (NOD/SCID) mice. Finally, we showed that IND treatment reduced the plasma activity and expression of autotaxin (ATX), the main LPA-producing enzyme, and also reduced the intestinal expression of *Lpar2* mRNA, which preceded the development of mucosal damage. We conclude that LPAR2 has a dual role in NSAID enteropathy, as it contributes to the maintenance of mucosal integrity after NSAID exposure, but also orchestrates the inflammatory responses associated with ulceration. Our study suggests that IND-induced inhibition of the ATX-LPAR2 axis is an early event in the pathogenesis of enteropathy.

## Introduction

Non-steroidal anti-inflammatory drugs (NSAIDs) belong to the most frequently used prescription and over-the-counter medications, being used by more than 30 million people on a daily basis [1]. The analgesic, anti-inflammatory and fever-reducing effects of NSAIDs rely on the ability of these drugs to inhibit the activity of cyclooxygenase (COX), and consequently, the production of inflammatory mediator prostaglandins. The chronic use of NSAIDs, however, is associated with several undesired effects, including damage to the gastrointestinal (GI) tract. NSAID-induced mucosal injury with inflammation and ulceration can occur in both the stomach and proximal duodenum (gastropathy), and in more distal parts of the small bowel (enteropathy), and is caused by the complex interplay of multiple factors [2–5].

NSAID-induced GI injury is likely to be initiated by the topical irritant effects of these drugs, including disruption of the membrane bilayer, epithelial mitochondrial dysfunction, leading to apoptosis [6, 7]. The reduced synthesis of prostaglandins, which otherwise play a key role in mucosal defence, aggravates the initial damage, and contributes to the disruption of mucosal barrier function [8, 9]. Increased mucosal permeability then allows the entry of luminal factors into the mucosa, which activate the innate arm of the immune system and recruit neutrophils to the mucosa. There is compelling evidence that activation of neutrophils, the release of reactive oxygen species, and proteolytic enzymes play a central role in NSAID-induced GI damage [10–13].

Although there is considerable overlap between the pathogenesis of NSAID-induced gastro- and enteropathy, there are also important differences. For example, the main luminal aggressors in gastropathy are gastric acid and pepsin, whereas in enteropathy, these are intestinal bacteria and bile. Consequently, NSAID enteropathy does not respond adequately to antisecretory drugs, which are the mainstay of NSAID gastropathy [14]. In fact, these drugs may even worsen NSAID-induced intestinal damage by changing the composition of gut microbiota [15]. Another approach to treat NSAID enteropathy is the use of mucoprotective agents, such as misoprostol, however, the high incidence of side effects may limit the use of this drug [16]. Therefore, there is a great need to find new therapeutic options for NSAID enteropathy that are both effective and have a favorable side-effect profile.

Lysophosphatidic acid (LPA) is a lipid mediator that regulates a wide variety of physiological and pathophysiological processes [17, 18]. It is produced from membrane phospholipids through two major metabolic pathways, either involving the hydrolysis of lysophospholipids by autotaxin (ATX) or the deacylation of phosphatidic acid [19]. LPA is present in all eukaryotic tissues examined, including the GI tract, where it can also originate from certain types of food, such as soy and cabbage [20].

LPA exerts its effects predominantly through six G protein-coupled receptors (LPAR1-6) [18]. All of them are expressed in the mouse small and large intestine [21, 22], with LPAR1 and LPAR5 displaying the highest levels [21]. Although the expression of LPAR2 is considerably lower, at least in the healthy gut, currently, much more information is available on the GI effects of LPAR2 than on those of other LPARs. For example, several studies have shown that LPAR2 activates prosurvival signaling pathways in intestinal epithelial cells and inhibits multiple steps of the apoptotic pathways, including the activation of caspase-3, which protects against irradiation-induced GI injury in mice [23–26]. In addition, LPAR2 activation mitigated irradiation-induced colonic mucosal barrier dysfunction [27], and also aspirin-induced gastric mucosal damage in mice [28]. The latter effect is presumably due to enhancing the production of mucoprotective prostaglandins [29, 30].

LPAR2 activation, on the other hand, may also contribute to pathological conditions, such as inflammation and tumorigenesis. LPAR2 expression is upregulated in gastric and colon cancers [31, 32], and also in dextrane sulfate sodium (DSS)-induced murine colitis [22, 33], and inhibition of LPAR2 attenuates both colitis and tumor formation [22, 34]. Hence, LPAR2 may have both protective and detrimental effects in the GI tract, depending on the applied model and disease context.

Since NSAID enteropathy is initiated by epithelial injury and loss of mucosal barrier function, which were effectively inhibited by LPAR2 activation in other disease models, and LPAR2 activation also mitigated NSAID gastropathy, we hypothesized that LPAR2 may be a promising target for the treatment of NSAID-induced intestinal damage. To test this hypothesis, we assessed the effects of LPAR2 deletion and activation on the development of indomethacin-induced small bowel injury in mice.

## Materials and methods

### Animals

Eight to ten-week-old male C57BL/6 mice (Charles River Laboratories, Isaszeg, Hungary) were used as wild-type (WT) animals. Mice deficient in the LPAR2 (*Lpar2*^*−/−*^) were generated and kindly provided by Dr. Jerold Chun (Sanford Burnham Prebys Medical Discovery Institute, USA) [35] and have been maintained with constant backcrossing to C57BL/6 since 2008. Eight to ten-week-old male NOD/SCID (non-obese diabetic/severe combined immunodeficiency) mice were obtained from the animal facility of the Department of Pathology and Experimental Cancer Research of Semmelweis University.

Animals were housed in a temperature (22 ± 2 °C)- and humidity-controlled room at a 12-h light/dark cycle with food and water available *ad libitum*. All procedures conformed to the Directive 2010/63/EU on European Convention for the protection of animals used for scientific purposes. The experiments were approved by the National Scientific Ethical Committee on Animal Experimentation and permitted by the government (Food Chain Safety and Animal Health Directorate of the Government Office for Pest County (PE/EA/1118-6/2020)).

### Study design and induction of enteropathy

To explore the effects of LPAR2 activation or deletion on NSAID enteropathy, we used a well-established model [13, 36, 37], in which a single, large dose (20 or 30 mg/kg) of indomethacin (IND), a non-selective COX inhibitor (Sigma, St. Louis, MO, USA) was given to mice via gastric gavage.

**Fig. 1:**
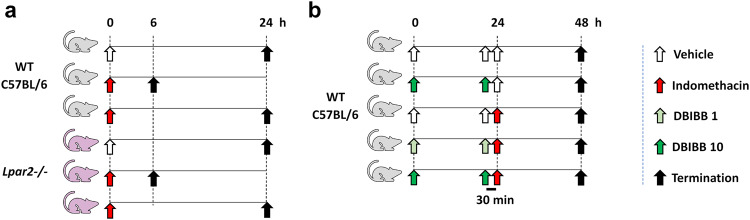
Experimental protocols. **a** Wild-type C57BL/6 (WT) and *Lpar2*^*−/−*^ mice were treated with IND (20 mg/kg) or its VEH (1% hydroxyethylcellulose) and euthanized after either 6 or 24 h. **b** WT mice were treated with IND (20 mg/kg) or its VEH and euthanized after 24 h. DBIBB (selective agonist of LPAR2, 1 or 10 mg/kg) or its VEH were given via gavage twice, 24 h and 30 min before the administration of IND.

In the first experiment, 24 WT and 19 *Lpar2*^*−/−*^ mice were randomly divided into three groups, with 6–8 animals in each group. The first group of WT and *Lpar2*^*−/−*^ mice received the vehicle (VEH) of IND via gastric gavage (VEH, 1% hydroxyethylcellulose; Sigma, St. Louis, MO, USA) and was euthanized 24 h later. The second and third groups were treated with 20 mg/kg IND and euthanized at 6 and 24 h postexposure (Fig. 1a).

In the second experiment, 35 WT animals were divided into five groups. The first group (“Control”) received only drug solvents. The second group (“DBIBB 10”) was treated with 10 mg/kg of DBIBB (2-[[[4-(1,3-dioxo-1H-benz[de]isoquinolin-2(3H)-yl)butyl]amino]sulfonyl]-benzoic acid) (Cayman Chemical, Ann Arbor, MI, USA), a selective agonist of LPAR2 [25], and then with the solvent of IND. The third group (“IND 20”) received the solvent of DBIBB (4% ethanol/14% propandiol in phosphate buffered saline), and then IND (20 mg/kg). The fourth and fifth groups were treated with both IND (20 mg/kg) and DBIBB (1 or 10 mg/kg) (“IND 20 + DBIBB 1” and “IND 20 + DBIBB 10”). DBIBB and its solvent were given via gastric gavage twice, 24 h and 30 min before the administration of IND or its VEH. The applied doses of DBIBB and the protocol of repeated daily administration were based on literature data [25, 38]. All groups were euthanized 24 h after the IND challenge (Fig. 1b).

In the third experiment, 32 NOD/SCID mice were used with the same protocol as in the second one (with 6–7 mice in each group) (Fig. 1b), except that IND was given at the dose of 30 mg/kg, because in our preliminary studies, these mice developed milder enteropathy than C57BL/6 mice.

At the time of sacrifice, plasma samples were collected and small intestines were excised. The length of the whole small intestine was measured, as one parameter to assess intestinal inflammation. Then, full-thickness pieces of the distal small intestine were snap-frozen in liquid nitrogen and stored at −80 °C for further analyses. A further distal segment was fixed in 10% formalin for evaluation of microscopic damage.

### Western blot analysis

Distal jejunal tissues were homogenized with a TissueLyser (Qiagen, Venlo, Netherlands) in lysis buffer supplemented with a protease inhibitor cocktail (cOmplete ULTRA Tablets, Roche, Basel, Switzerland) and PMSF (Sigma, St. Louis, MO, USA). The homogenates were centrifuged twice at 1500 × *g* and 4 °C for 15 min and the supernatants were collected, their protein concentration was measured by the bicinchoninic acid assay (BCA, Thermo Fisher Scientific, Waltham, MA, USA). Equal amount of protein (20 µg) was mixed with Pierce Lane Marker reducing sample buffer (Thermo Fisher Scientific, Waltham, MA, USA), and loaded and separated in a 4%–20% precast Tris-glycine SDS polyacrilamide gel (BioRad, Hercules, CA, USA). Proteins were transferred electrophoretically onto a polyvinylidene difluoride membrane (BioRad, Hercules, CA, USA) at 200 mA overnight. Membranes were blocked with 5% nonfat dry milk (BioRad, Hercules, CA, USA) in Tris-buffered saline containing 0.05% Tween-20 (0.05% TBS-T; Sigma, St. Louis, MO, USA) at room temperature for 2 h. Membranes were incubated with primary antibodies against COX-2 (#12282, 1:500), COX-1 (#4841, 1:500), cleaved caspase-3 (#9664, 1:1000), caspase-3 (#9662, 1:1000) (all from Cell Signaling Technology, Danvers, MA, USA), interleukin-1β (IL-1β, ab9722, 1:1000), pentraxin-3 (PTX3, ab125007, 1:1000), tumor necrosis factor-α (TNF-α, ab66579, 1:1000) (Abcam, Cambridge, UK), myeloperoxidase (MPO, AF3667, 1:1000, R&D Systems, Minneapolis, MN, USA) and cathelicidin antimicrobial peptide (CAMP, PAC419Ra01, 1:1000, Cloud-Clone, Katy, TX, USA) overnight at 4 °C, followed by 2 h incubation at room temperature with an appropriate HRP-linked secondary antibody. GAPDH (D16H11, 1:1000, Cell Signaling Technology, Danvers, MA, USA) was used to control for sample loading and protein transfer and to normalize the content of target protein. Membranes were trimmed before the antibody treatment if the bands of interest were far apart. At least two repetitions were performed for each experiment. Signals were detected with a chemiluminescence kit (BioRad, Hercules, CA, USA) by Chemidoc XRS+ (BioRad, Hercules, CA, USA).

The protein amount of ATX (anti-ATX, 10005375, 1:250, Cayman Chemical, Ann Arbor, MI, USA) was measured from the plasma. The analysis was performed as described above, except that 40 µg of plasma protein was loaded in the wells of the gel and albumin was used as loading control (sc-271605, 1:5000, Santa Cruz Biotechnology, Santa Cruz, CA, USA).

### Histological analysis

Samples taken from the distal part of the small intestine were fixed in 10% formalin, embedded in paraffin, sectioned (4 µm), and stained with hematoxylin and eosin. The severity of epithelial damage, mucosal and submucosal edema, and neutrophil infiltration was assessed by a histopathologist blinded to the interventions by using a scoring system described previously [39], with some modifications. Each parameter was scored from 0 (no alterations) to 3 (presence of ulcers, widely spaced crypts with numerous red blood cell-containing vessels in lamina propria, and numerous neutrophils in the lamina propria), and the total histological score was calculated based on the sum of partial scores. Representative images were captured with a Leica LMD6 microscope (Leica, Wetzlar, Germany).

### Immunohistochemistry

Small intestinal tissues were fixed in 10% neutral buffered formalin and embedded in paraffin. Approximately 2.5 µm thick serial sections were cut and processed for immunohistochemistry. For antigen retrieval, sections were heated for 20 min in Tris-EDTA buffer pH 9.0 (0.1 M Tris base and 0.01 M EDTA) followed by a 20 min cooling. Endogenous peroxidases were blocked using 3% H_2_O_2_ in methanol, while non-specific proteins were blocked with 3% bovine serum albumin (BSA, #82-100-6, Millipore, Kankakee, IL, USA) diluted in 0.1 M Tris-buffered saline (TBS, pH 7.4) containing 0.01% sodium-azide, both for 15 min. The sections were incubated with MPO primary antibody (AF3667, 1:900, R&D Systems, Minneapolis, MN, USA) diluted in 1% BSA/TBS + TWEEN (TBST, pH 7.4) overnight (16 h) in a humidified chamber. Peroxidase conjugated donkey anti-goat IgG (ab214881, Abcam, Cambridge, UK) was used for 40 min incubation and the enzyme activity was revealed by 3,3′-diaminobenzidine (DAB) chromogen/hydrogen peroxide kit (DAB Quanto, #TA-060-QHDX, Thermo, WA, USA) under microscopic control. All incubations were done at room temperature, with samples washed between incubations in TBST buffer for 5 min twice. Digitalization of slides was done using modules of the QuantCenter image analysis software tool pack (3DHISTECH, Budapest, Hungary). The number of MPO-positive cells was counted in at least 20 randomly selected villi from a single Swiss-roll section of each animal.

### qRT-PCR measurements

Total RNA was obtained from 10 to 30 mg of small intestine tissue using the QIAzol extraction method (Qiagen, Hilden, Germany). RNA concentration was measured with a Nanophotometer (Implen GmbH, Munich, Germany). Reverse transcription was performed from 1 μg of total RNA with a Sensifast cDNA synthesis kit (Bioline, London, UK) according to the manufacturer’s protocol. Target genes were amplified using a LightCycler^®^ 480 II instrument (Roche, Germany) using the SensiFAST SYBR Green master mix (Bioline, UK). Expression levels were calculated with the 2^–ΔΔCT^ evaluation method and *Rpl13a* or *Rplp0* was used as reference genes. The sequences of primers used for determination are listed in Table 1.

**Table 1:** List of primer sequences.

Gene	Primer sequence (5′-3′)		Accession number
*Enpp2*	forward	ATGTGCGATCTCCTAGGCTTG	NM_001411653.1
	reverse	ACCTTATCATCACAGGTGCAG	
*Lpar2*	forward	ATGTGCGTAGACGGGTGGAAC	NM_020028.3
	reverse	TGAGTGTGGTCTCTCGGTAGC	
*Rpl13a*	forward	GGATCCCTCCACCCTATGACA	NM_173340.2
	reverse	CTGGTACTTCCACCCGACCTC	
*Ptx3*	forward	CTGCCCGCAGGTTGTGAAA	NM_008987.3
	reverse	ACAGGATGCACGCTTCCAAA	
*Tnf-α*	forward	TAGCCCACGTCGTAGCAAAC	NM_013693.3
	reverse	ACAAGGTACAACCCATCGGC	
*Rplp0*	forward	CTCTCGCTTTCTGGAGGGTG	NM_007475.5
	reverse	ACGCGCTTGTACCCATTGAT	

### Intestinal cytokine/chemokine analysis

The cytokine and chemokine profile of small intestinal samples was determined by a Proteome Profiler Mouse Cytokine Array Kit Panel A (ARY006, R&D Systems, Minneapolis, MN, USA) according to the manufacturer’s instructions. Bound antibodies were quantified using chemiluminescence (Chemidoc XRS+, BioRad, Hercules, CA, USA). The intensity of the emitted light at each spot was analyzed by densitometry (Image Lab Software, Bio-Rad, Hercules, CA, USA) and normalized to the pixel density of reference spots (10,000) [40].

### RNAscope in situ hybridization

RNAscope in situ hybridization assay was performed on small intestine tissue slides using RNAscope Multiplex Fluorescent Kit v2 according to the manufacturer’s instructions (Advanced Cell Diagnostics, Newark, CA, USA). Briefly, 4 µm formalin-fixed paraffin-embedded tissue sections were pretreated with heat and protease prior to hybridization with the following target oligo probes: 3plex-Positive Control Probe-Mm (catalog number: 320881), 3plex-Negative Control Probe (catalog number: 320871) and Mm-Lpar2 (catalog number: 442691, accession number: NM_020028.3). Preamplifier, amplifier, and AMP-labeled oligo probes were then hybridized sequentially, followed by signal development with TSA fluorophores (TSA-Cy3, Akoya Biosciences, Marlborough, MA, USA). Each sample was quality controlled for RNA integrity with a positive control probe specific to housekeeping genes and with a negative control probe set. The pretreatment conditions were optimized to establish the maximum signal-to-noise ratio. Specific RNA staining signal was identified as red punctate dots. Nuclei were stained with 4′,6-diamidino-2-phenylindole (DAPI). Imaging was performed with Leica DMI8 Confocal microscope.

*Lpar2* signal was quantified by using the subcellular spot detection command of QuPath (open-source software, available at https://qupath.github.io/) [41] and was expressed as dots/1000 μm^2^.

### Measurement of plasma ATX activity

Heparin-anticoagulated plasma (10 μL) of VEH- and IND-treated mice was incubated with 2 μM FS-3 ATX substrate (Echelon Biosciences, Salt Lake City, USA) and 10 μM BSA in a total 60 μL of assay buffer consisting of 50 mM TRIS, 140 mM NaCl, 5 mM KCl, 1 mM CaCl_2_, and 1 mM MgCl_2_ (pH 8.0) for 4 h at 37 °C. The fluorescence (λ_excitation_ = 494 nm and λ_emission_ = 520 nm) was recorded every 2 min by a Varioskan™ LUX Multimode Microplate Reader for 240 min (Thermo Fisher Scientific, Waltham, MA, USA). Linear reaction rates were reported as ATX activity in terms of relative fluorescence units (RFU) / min.

In order to assess the direct effect of IND on plasma ATX activity the same protocol was used as above, but only the plasma from VEH-treated mice was used and spiked with IND (30–300 μM) or its VEH. This concentration range was chosen based on previous pharmacokinetic studies showing that the peak plasma concentration (*C*_max_) of IND is 76 μg/mL (212 μM) after a single, 20 mg/kg oral dose in mice [42], and its half-life is around 10 h [36].

### Measurement of human recombinant ATX activity

First, we used the same FS-3 assay as described above, but instead of plasma 10 μL of human recombinant ATX (3, 10 and 30 nM) (Daresbury Proteins Ltd, Warrington, UK) was incubated with FS-3 substrate ± IND (30–300 μM).

In addition, the effect of IND on human recombinant ATX-induced hydrolysis of LPC 18:1 was assessed with the Amplex Red choline release assay. ATX was generated in-house, as described before [43]. Triplicate wells were loaded with 60 μL of reaction cocktail in ATX assay buffer (50 mM TRIS, 150 mM NaCl, 5 mM CaCl_2_, 30 μM BSA, pH 7.4), with of 10 μM Amplex Red, 1 U/mL horeseradish peroxidase (both from Thermo Fisher Scientific, Waltham, MA, USA) and 0.1 U/mL choline oxidase (MP Biomedicals, Irvine, CA, USA), resulting in an overall concentration of 100 μM LPC 18:1 (Avanti Polar Lipids, Alabaster, AL, USA) with 10 nM ATX ± IND (30–300 μM). The fluorescence (λ_excitation_ = 560 nm and λ_emission_ = 590 nm) was recorded every 2 min by a FlexStation 3 Multi-Mode Microplate Reader for 240 min (Molecular Devices, San Jose, CA, USA).

### Statistical analysis

Statistical analysis of the data was performed with Student *t* test, one-way ANOVA or two-way ANOVA, followed by Fisher’s LSD *post hoc* test, or in case of nonparametric values Kruskal–Wallis test followed by uncorrected Dunn’s *post hoc* test. Correlations between the expression of cleaved caspase-3 and histological scores were calculated by Spearman test. Outliers detected by Grubb’s test were excluded from the analyses. A probability of *P* < 0.05 was considered statistically significant.

## Results

### IND-induced intestinal damage and inflammation shows an early onset in *Lpar2*^*−/−*^ mice

**Fig. 2:**
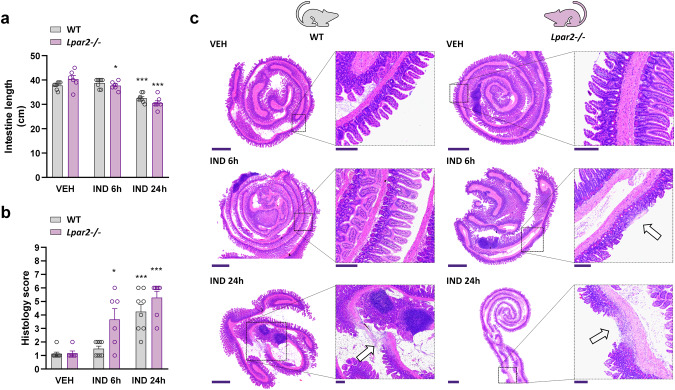
*Lpar2*^*−/−*^ mice are more susceptible to IND-induced enteropathy than wild-type mice. IND (20 mg/kg) or its VEH were administered by gavage to wild-type (WT) and *Lpar2*^*−/−*^ mice, and enteropathy-induced shortening of the small intestine (**a**) and histological injury (**b**, **c**) were assessed after 6 and 24 h. **a**, **b**: Circles represent the data of each mouse, bars indicate the mean + SEM. **c** Representative histological images (haematoxylin and eosin staining, low magnification scale bar: 1000 μm, high magnification scale bar: 200 μm) of the small intestines of IND-treated WT and *Lpar2*^*−/−*^ mice, arrows mark mucosal lesions, characterized by epithelial loss and inflammatory infiltration. For statistical analysis two-way ANOVA (**a**) and Kruskal–Wallis test (**b**) were used, followed by Fisher’s LSD and uncorrected Dunn’s tests, respectively. *n* = 6–8/group, *P < 0.05, ***P < 0.001 compared to the respective VEH-treated group.

In the present study, a 20 mg/kg dose of IND induced mild enteropathy within 24 h in WT mice. Although there were no visible signs of mucosal damage in any of the mice, the length of small intestine decreased after 24 h, indicative of inflammation (Fig. 2a). Histological examination revealed the presence of erosions, mild edema and infiltration of neutrophils, resulting in higher overall histology score in IND-treated than in VEH-treated mice (Fig. 2b, c). Neither shortening of the intestine nor histological changes were observed 6 h after IND treatment in WT animals.

In comparison, in *Lpar2*^*−/−*^ mice shortening of the intestine, development of mucosal erosions and tissue infiltration of neutrophils started earlier, as early as 6 h after the administration of IND, suggesting that deletion of *Lpar2* promoted the development of enteropathy. After 24 h there were no significant differences in terms of intestine shortening and histological damage between WT and *Lpar2*^*−/*−^ mice.

### Earlier neutrophil activation is accompanied by lower levels of other inflammatory mediators in enteropathy of *Lpar2*^*−/−*^ mice than in WT mice

Next, we aimed to characterize the inflammation in intestinal samples. We focused on inflammatory markers produced mainly by epithelial cells, macrophages, and neutrophils, because based on previous studies the adaptive immune system does not play a critical role in the development of NSAID enteropathy [12, 44].

**Fig. 3:**
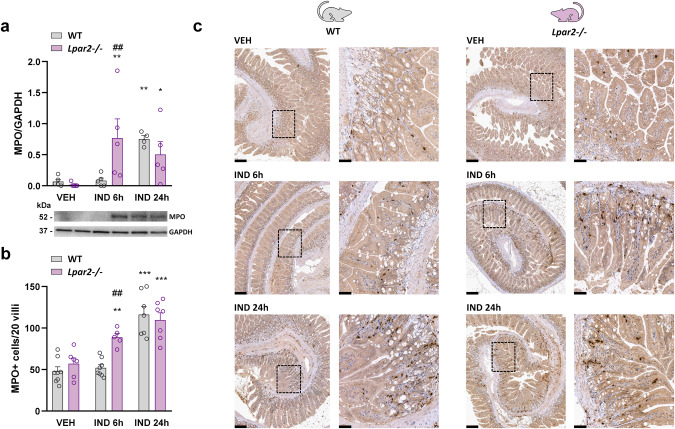
Neutrophil recruitment into the mucosa starts earlier in *Lpar2*^*−/−*^ mice compared to wild-type mice. The effect of IND (20 mg/kg) or its VEH on the tissue level of MPO protein (**a**), and on the number of MPO+ cells in the small intestinal villi (**b**, **c**) of wild-type (WT) and *Lpar2*^*−/−*^ mice. **a**, **b** Circles represent the data of each mouse, bars indicate the mean + SEM. **c** Representative images of MPO staining (low magnification: scale bar 200 μm, high magnification: scale bar 50 μm). For statistical analysis two-way ANOVA was used, followed by Fisher’s LSD test. *n* = 4–7/group, *P < 0.05, **P < 0.01, ***P < 0.001 compared to the respective VEH-treated group, ^##^*P* < 0.01 compared to the respective WT group.

First, we assessed the tissue level of the neutrophil marker MPO by Western blotting and immunohistochemistry (Fig. 3). We found that both MPO protein expression and the mucosal count of MPO positive cells increased 6 h after IND treatment in *Lpar2*^*−/−*^ mice, but not in WT mice, supported by the results of histological analysis. On the other hand, after 24 h MPO levels were similarly high in both cohorts.

**Fig. 4:**
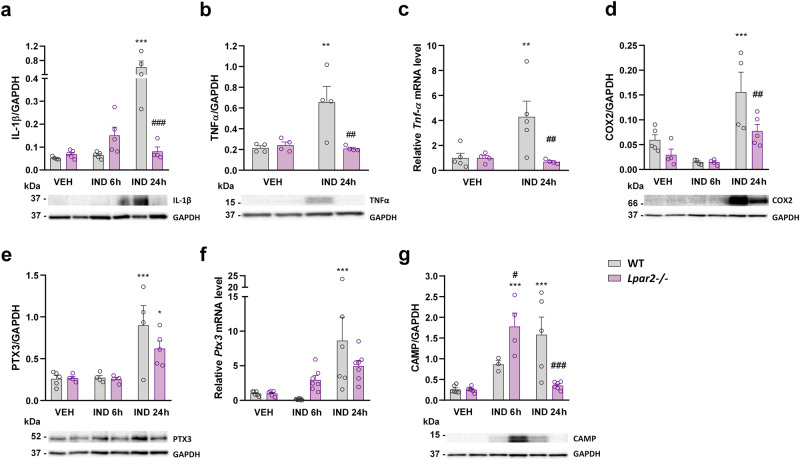
The tissue levels of inflammatory mediators are lower in *Lpar2*^*−/−*^ mice compared to wild-type mice at 24 h after IND treatment. The effect of IND (20 mg/kg) or its VEH on the tissue levels of different inflammatory mediators. Circles represent the data of each mouse, bars indicate the mean + SEM. For statistical analysis, two-way ANOVA was used, followed by Fisher’s LSD test. *n* = 3–7/group, *P < 0.05, **P < 0.01, ***P < 0.001 compared to the respective VEH-treated group, ^##^*P* < 0.01, ^###^*P* < 0.001 compared to the respective WT group.

It is well-established that mucosal recruitment of neutrophils in NSAID enteropathy is preceded by the release of proinflammatory cytokines, such as IL-1β, from the small intestinal epithelial cells and macrophages [11, 45]. Here, we found that IL-1β expression was slightly higher in *Lpar2*^*−/*−^ than in WT mice at 6 h but ~8-times lower at 24 h (Fig. 4a). The expression of IL-1β at this time point was comparable to those of VEH-treated mice. We assessed the protein and mRNA levels of TNF-α, another key cytokine in enteropathy-associated inflammation [11, 45, 46]. We found low levels of this cytokine in *Lpar2*^*−/−*^ mice 24 h after IND treatment (Fig. 4b, c), despite that histological signs of mild injury and neutrophil activation were present.

The upregulation of the inducible COX-2 enzyme in response to various proinflammatory stimuli, including the cytokines IL-1β and TNF-α [47], is a hallmark of NSAID-induced GI injury. COX-2-derived prostaglandins amplify the inflammatory response, however, they also contribute to the maintenance of mucosal integrity [48, 49]. We found low levels of COX-2 in VEH-treated WT mice, and even slightly lower levels in *Lpar2*^*−/−*^ mice. IND treatment resulted in a 3-fold elevation of COX-2 expression after 24 h, but this effect was reduced in *Lpar2*^*−/−*^ mice (Fig. 4d). The measurement of pentraxin-3, another contributor of innate immunity [50], yielded similar results (Fig. 4e, f).

Finally, we assessed the expression of the cathelicidin antimicrobial host defence peptide (CAMP), which is known to be upregulated during inflammation and wound healing [51]. Although the intestinal level of CAMP increased in both WT and *Lpar2*^*−/−*^ mice after IND treatment, it had a different time course in the two strains. Namely, it increased gradually in WT mice, whereas showed more rapid elevation in *Lpar2*^*−/*−^ mice but returned to normal levels after 24 h (Fig. 4g).

Taken together, tissue inflammation associated with enteropathy developed more rapidly in the absence of LPAR2, in particular, due to earlier recruitment and activation of neutrophils. The lower levels of most inflammatory mediators measured, however, indicate that deletion of *Lpar2* interfered with several components of the inflammatory response.

### LPAR2 stimulation reduces IND-induced mucosal damage, but aggravates inflammation

In the next step, we aimed to determine the effect of DBIBB, a selective LPAR2 agonist [25], on IND-induced small intestinal damage. Since the lack of LPAR2 promoted the development of enteropathy, DBIBB was expected to induce mucosal protection, and the severity of IND-induced damage was assessed only at a later time point, 24 h post-dose.

**Fig. 5:**
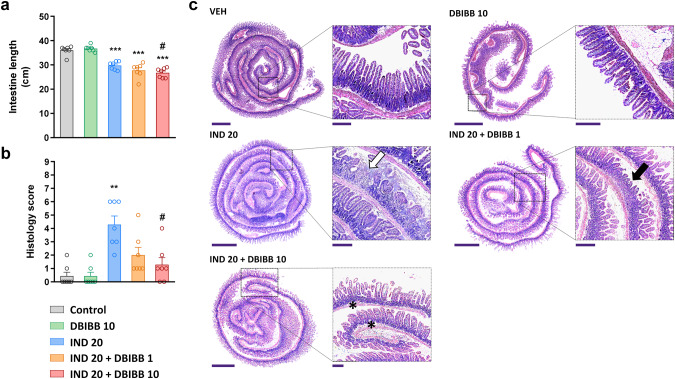
The selective LPAR2 agonist DBIBB increases the shortening of small intestine but reduces the severity of mucosal damage in IND-treated mice. DBIBB (1 and 10 mg/kg) or its VEH were administered by gavage twice, 24 h and 30 min before the administration of IND (20 mg/kg). The length of small intestines (**a**) and histological injury (**b**, **c**) were assessed 24 h after IND treatment. **a**, **b** Circles represent the data of each mouse, bars indicate the mean + SEM. **c** Representative histological images (haematoxylin and eosin staining, low magnification scale bar: 1500 μm, high magnification scale bar: 250 μm) of the small intestines, white and black arrows denote ulcer and superficial epithelial damage, respectively, whereas asterisks denote mucosal edema. For statistical analysis one-way ANOVA (**a**) and Kruskal–Wallis test (**b**) were used, followed by Fisher’s LSD and uncorrected Dunn’s tests, respectively. *n* = 7/group, **P < 0.01, ***P < 0.001 compared to control (only VEH-treated) group, ^#^*P* < 0.05 compared to “IND 20” group.

As in the first experiment, IND did not cause visible damage to the small intestinal mucosa, but induced shortening of the bowel and various histological alterations characteristic for enteropathy, such as focal ulcerations, edema, and neutrophil infiltration (Fig. 5). DBIBB alone (10 mg/kg) did not cause any macroscopic or histological changes. When combined with IND, DBIBB at lower dose (1 mg/kg) tended to increase IND-induced bowel shortening (*P* = 0.07), whereas at higher dose (10 mg/kg) increased it, suggesting that inflammation was more pronounced after LPAR2 stimulation (Fig. 5a). Interestingly, histological analysis revealed less epithelial damage and reduced neutrophil infiltration in IND + DBIBB-treated mice, resulting in reduced composite histological scores compared to the animals treated only with IND (Fig. 5b, c).

**Fig. 6:**
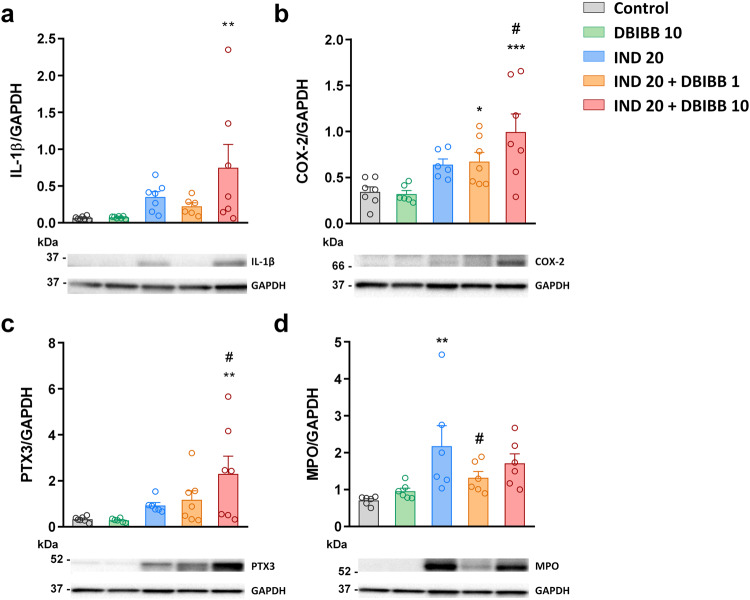
The selective LPAR2 agonist DBIBB increases the IND-induced elevation of different inflammatory mediators, but not that of myeloperoxidase. DBIBB (1 and 10 mg/kg) or its VEH were administered by gavage twice, 24 h and 30 min before the administration of IND (20 mg/kg), the protein expression of inflammatory mediators was assessed by Western blotting. Circles represent the data of each mouse, bars indicate the mean + SEM. For statistical analysis, one-way ANOVA was used, followed by Fisher’s LSD test. *n* = 6–7/group, **P < 0.01, ***P < 0.001 compared to control (only VEH-treated) group, ^#^*P* < 0.05 compared to “IND 20” group.

Western blot analysis of innate immunity-related inflammatory markers (Fig. 6) showed that DBIBB treatment alone had no effect on the expression of any of them, but it potentiated the elevation of IL-1β and PTX3 in animals with enteropathy. DBIBB also increased the IND-induced elevation of COX-2 expression in a dose-dependent manner. Of note, expression of the constitutive COX-1 enzyme remained unaltered in all treatment groups (data not shown). We also assessed the expression of MPO, which rather decreased due to DBIBB treatment in enteropathy, confirming the histological finding of reduced neutrophil infiltration in these groups.

Collectively, these results indicate that pharmacological stimulation of the LPAR2 reduced the severity of IND-induced intestinal damage, but at the same time, it also promoted the development of tissue inflammation. The lower neutrophil scores and MPO levels, however, suggest that the increased inflammatory reaction in DBIBB-treated mice was not driven primarily by neutrophils.

### DBIBB increases the levels of numerous chemokines and cytokines in the inflamed intestinal tissue, including those released by T cells

**Fig. 7:**
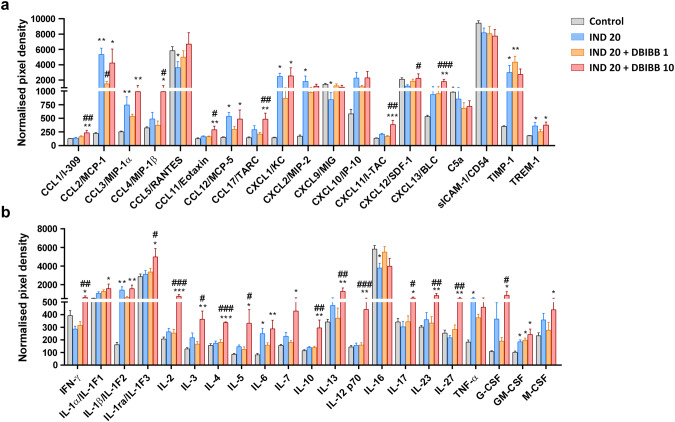
The selective LPAR2 agonist DBIBB increases the IND-induced elevation of several proinflammatory chemokines and cytokines. DBIBB (1 and 10 mg/kg) or its VEH were administered by gavage twice, 24 h and 30 min before the administration of IND (20 mg/kg), the protein expression of proinflammatory mediators was assessed by a cytokine/chemokine array kit. Bars indicate the mean + SEM. For statistical analysis, one-way ANOVA was used, followed by Fisher’s LSD test. *n* = 4–6/group, ^*^P < 0.05, ^**^P < 0.01, ^***^P < 0.001 compared to control (only VEH-treated) group, ^#^*P* < 0.05, ^##^*P* < 0.01, ^###^*P* < 0.001 compared to “IND 20” group.

We aimed to characterize the inflammatory reaction in more detail by using a mouse cytokine/chemokine array kit. Because DBIBB alone had no effect on any of the previously measured parameters, proteome analysis in this group was not performed. As Fig. 7 demonstrates, IND treatment increased the levels of numerous cytokines and chemokines, mainly originating from epithelial cells, mast cells, macrophages, dendritic cells and/or neutrophils during an acute inflammatory state, including CCL2, CCL3, CCL12, CXCL1, CXCL2, TIMP-1, TREM-1, IL-1β, IL-6, TNF-α, and GM-CSF. Of note, cytokines secreted predominantly by lymphocytes were not increased by IND treatment.

The lower dose of DBIBB tended to reduce the levels of some inflammatory proteins compared to the IND-treated group, although only the reduction of CCL2 was statistically significant. In contrast, the higher dose of DBIBB increased the tissue levels of many chemokines and cytokines. Specifically, it raised the tissue concentrations of CCL1, CCL4, CCL11, CCL17, CXCL11, CXCL12, and CXCL13, that are chemokines secreted by multiple cell types, including macrophages, dendritic cells, endothelial and epithelial cells.

In addition, there was a remarkable elevation in the levels of several cytokines derived from T cells. There was no clear difference with regard to the type of secreting T cells, as both Th1- (IFN-γ, IL-2), Th2- (IL-4, IL-5, IL-10, IL-13), and Th17-associated cytokines (IL-17) were increased.

Taken together, these results confirm the proinflammatory effect of DBIBB in IND-induced enteropathy and suggest that cytokines and chemokines were released by multiple immune cells, including T cells.

### Lack of LPAR2 promotes caspase-3 activation in IND-induced enteropathy, whereas DBIBB treatment reduces it in NOD/SCID mice

**Fig. 8:**
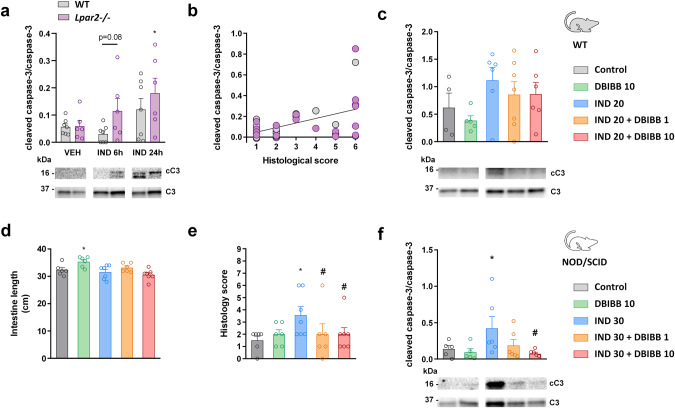
The lack of LPAR2 increases, whereas activation of LPAR2 by DBIBB reduces caspase-3 activation. **a** Tissue expression of total (C3) and cleaved caspase-3 (cC3) in wild-type (WT) and *Lpar2*^*−/*−^ mice treated with IND or VEH. **b** Correlation between caspase-3 activation and the severity of histological damage (R^2^ = 0.25, *P* = 0.02). **c** Caspase-3 activation in WT mice treated with DBIBB. **d**–**f** The effects of DBIBB treatment on small intestinal length, histological scores and caspase-3 activation in NOD/SCID mice. Circles represent the data of each mouse, bars indicate the mean + SEM. For statistical analysis two-way ANOVA (**a**), Spearman test (**b**), one-way ANOVA (**c**, **d**, **f**) and Kruskal–Wallis test (**e**) were used. *n* = 6–7/group, *P < 0.05 compared to respective control (only VEH-treated) group, ^#^*P* < 0.05 compared to “IND 30” group.

LPAR2 activation inhibits various steps of the apoptotic pathway, including the activation of caspase-3 [24–26], whereas the absence of this receptor promotes intestinal apoptosis [24, 52]. It has also been shown that increased caspase-3 activation and apoptosis contribute to IND-induced gastric and small intestinal injury [53, 54]. Hence, next, we determined the level of cleaved caspase-3 in the small intestine of WT and *Lpar2*^*−/−*^ mice. In WT mice IND had no effect on caspase-3 activation 6 h post-dose, whereas it induced modest, non-significant elevation of cleaved caspase-3 after 24 h (Fig. 8a). The absence of LPAR2 had no effect on caspase-3 activation in VEH-treated animals, however, it potentiated the proapoptotic effect of IND. Namely, the level of cleaved caspase-3 started to rise already at 6 h in IND-treated *Lpar2*^*−/*−^ mice and increased further after 24 h. These levels reflected to some extent the histological damage scores, and showed a weak but significant correlation with them (*R*^2^ = 0.25, *P* = 0.02) (Fig. 8b).

DBIBB treatment, on the other hand, had no major impact on caspase-3 activation in WT animals, although there was a trend toward reduction of cleaved caspase-3 in DBIBB-treated mice (Fig. 8c).

It is well-established that certain proinflammatory cytokines can promote intestinal epithelial apoptosis [55], and apoptosis is increased in inflammatory bowel disease [56]. Because the higher dose of DBIBB increased the tissue level of several cytokines in IND-treated WT mice, we hypothesized that the proinflammatory effects of DBIBB may mask its potential antiapoptotic properties, whereas suppression of the immune functions could reveal them. Therefore, we have evaluated the effect of DBIBB in IND-treated NOD/SCID mice, which lack functional T and B lymphocytes and also have impaired macrophage, dendritic and NK cell functions, whereas neutrophils and monocytes are functional [57].

In general, IND induced mild enteropathy in NOD/SCID mice. IND treatment caused neither macroscopic damage to the small intestinal mucosa nor shortening of the intestine length (Fig. 8d). Histological analysis revealed the presence of some erosions in IND-treated animals, and also mild mucosal edema with modest neutrophil infiltration, although these two latter morphological alterations were similarly observed in some of the control animals. DBIBB treatment reduced the overall histological score of indomethacin-treated animals (Fig. 8e), confirming the mucoprotective effect of LPAR2 stimulation. In addition, in immunodeficient mice DBIBB also reduced the expression of cleaved caspase-3 in a dose-dependent fashion (Fig. 8f).

These results suggest that the absence of LPAR2 accelerates the development of IND-induced enteropathy in part by increasing the rate of intestinal apoptosis. In contrast, DBIBB may reduce intestinal apoptosis, but this effect may be attenuated by its proinflammatory properties.

### IND treatment reduces both plasma ATX activity and intestinal *Lpar2* mRNA expression

**Fig. 9:**
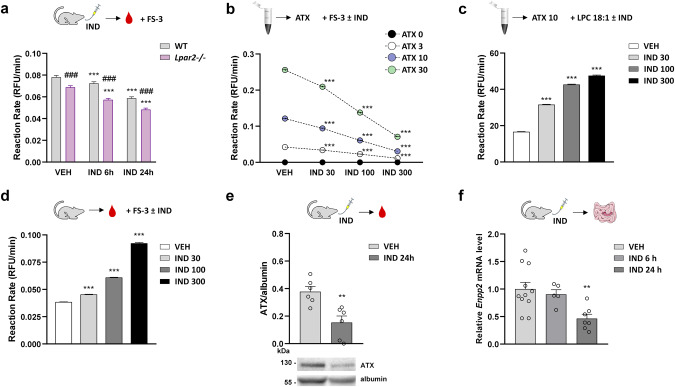
Autotaxin activity and expression are both reduced in IND-treated mice. The effect of IND on autotaxin (ATX) activity and expression. **a** Activity of ATX in the plasma of wild-type (WT) and *Lpar2*^*−/−*^ mice treated with either VEH or IND (20 mg/kg). **b** The effect of IND (30–300 μM) on FS-3 hydrolysis caused by human recombinant ATX (3–30 nM). **c** The effect of IND on LPC 18:1 hydrolysis caused by human recombinant ATX (10 nM). **d** FS-3 hydrolysis in plasma of VEH-treated mice spiked with IND. **e**, **f** Expression of ATX protein (plasma) and *Enpp2* mRNA (small intestine) in VEH- and IND-treated mice. Values indicate the mean + SEM, circles on (**e**) and (**f**) represent the data of each mouse. For statistical analysis two-way ANOVA (**a**), one-way ANOVA followed by Fisher’s LSD test (**b**–**d**, **f**), or Student *t* test (**e**) were used, *n* = 3–7/group, **P < 0.01, ***P < 0.001 compared to VEH-treated group, ^###^*P* < 0.001 compared to the respective WT group.

Finally, we explored the effects of IND treatment on the activity of ATX, a main source of LPA in serum and plasma [19]. We found that plasma ATX activity, measured by the hydrolysis of the synthetic substrate FS-3, was lower in VEH-treated *Lpar2*^−*/−*^ mice than in WT mice and IND induced a time-dependent reduction in plasma ATX activity in both groups (Fig. 9a). We wanted to determine whether this is due to direct inhibition of ATX by IND or to decreased ATX expression, therefore, we tested the effect of IND on ATX activity at concentrations of 30–300 μM by using different assays, however, these studies have yielded inconclusive results. Namely, direct addition of IND decreased the hydrolysis of FS-3 by human recombinant ATX (Fig. 9b), whereas increased the ATX-mediated hydrolysis of LPC 18:1 assessed by the Amplex Red choline release assay (Fig. 9c). When the plasma of VEH-treated mice was spiked with IND, we found a concentration-dependent increase in ATX-mediated FS-3 hydrolysis (Fig. 9d). These results suggest that high concentrations of IND have variable interfering effects on ATX activity depending on the assay used. Nevertheless, it is unlikely that the decreased plasma ATX activity observed in IND-treated mice is caused by direct inhibition of ATX, as IND rather increased than decreased ATX activity at the concentrations tested.

On the other hand, we found that the level of ATX protein in plasma and the small intestinal expression of the *Enpp2* gene, encoding ATX, were both reduced in IND-treated mice (Fig. 9e, f), suggesting that reduced plasma ATX activity in IND-treated mice is mainly due to downregulation of ATX expression.

**Fig. 10:**
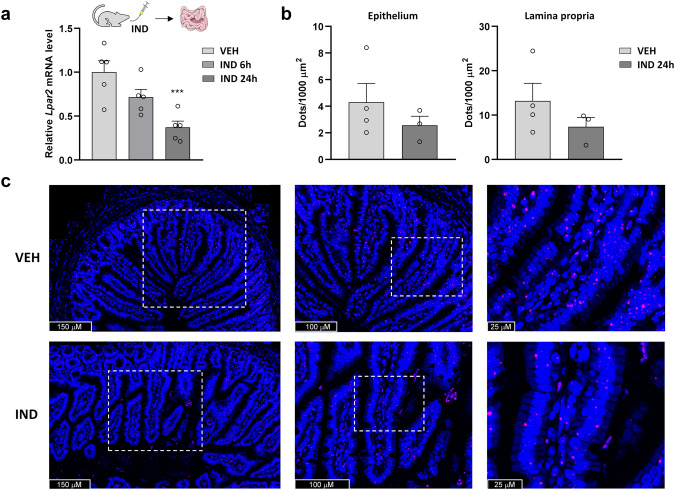
Intestinal expression of *Lpar2* is reduced in IND-treated mice. The effect of IND (20 mg/kg) treatment on the small intestinal expression of *Lpar2* mRNA measured by qPCR (**a**) and RNAscope (**b**). Bars indicate the mean + SEM, circles represent the data of each mouse. For statistical analysis either one-way ANOVA followed by Fisher’s LSD test (**a**) or Student *t* test (**b**) was used, *n* = 3–7/group, ***P < 0.001 compared to VEH-treated group. **c** Representative confocal microscopy images of RNAscope-*Lpar2* expression in the small intestine of VEH- and IND-treated mice. Nuclei were stained with DAPI (blue). Cy3-labeled tyramide (red) was used to visualize *Lpar2*.

Similarly, IND decreased the intestinal level of *Lpar2* mRNA in WT mice (Fig. 10a). The finding that downregulation of ATX activity and *Lpar2* expression both started already 6 h after IND treatment in WT mice suggests that suppression of the ATX-LPA-LPAR2 axis precedes the development of mucosal damage and inflammation. We also aimed to determine the localization of LPAR2 in the small intestine. Because we did not find reliable antibody against LPAR2, we performed RNA scope in situ hybridization assay to visualize the mRNA of *Lpar2*, and we found that it is located in both the epithelium and lamina propria of control and IND-treated animals. Quantification of the *Lpar2* signal confirmed the qPCR result that *Lpar2* gene expression is reduced in the small intestine of IND-treated mice and extended it by showing that this reduction occurs in both the epithelium and lamina propria cells (Fig. 10b, c).

## Discussion

Our study provides the first demonstration that LPAR2 has a role in maintaining intestinal mucosal integrity after NSAID exposure-induced enteropathy and regulates the inflammatory response associated with ulceration. Therefore, selective LPAR2 activation has both mucoprotective and proinflammatory effects in the context of NSAID enteropathy. We also showed that IND inhibits plasma ATX activity and downregulates the small intestinal expression of *Lpar2*, which precede and potentially lead to the development of mucosal damage. Hence, inhibition of the ATX-LPAR2 axis may be an early event in the pathogenesis of IND-induced enteropathy.

There is compelling evidence that LPA, a growth factor-like lipid mediator, plays a crucial role in the protection of GI mucosa from a variety of noxious conditions [23, 24, 28, 58–61]. The development and use of novel, highly selective LPAR ligands and targeted gene knockout animal models have also allowed to identify LPAR2 as a central regulator of protection against γ-irradiation-induced intestinal injury [24, 25], NSAID-induced gastropathy [28] and toxin-induced diarrheas [62]. Our results provide further evidence for the mucosal protective property of LPAR2 and demonstrate for the first time that selective activation of LPAR2 by DBIBB mitigates NSAID-induced small intestinal injury, whereas lack of LPAR2 accelerates the development of enteropathy. The higher sensitivity of *Lpar2*^*−/−*^ mice against IND suggests that LPAR2 may have a role in maintaining the integrity of small intestinal mucosa exposed to NSAIDs.

One of the earliest factors contributing to NSAID-induced GI injury is the topical damaging effect of these drugs, which involves epithelial apoptosis due to mitochondrial dysfunction and endoplasmic reticulum stress [6, 7, 53, 63]. The importance of increased epithelial apoptosis has also been demonstrated in the pathogenesis of IND-induced small intestinal injury [54, 64]. By measuring the tissue level of cleaved caspase-3, the main effector caspase in the apoptotic pathway [65], we confirmed the proapoptotic effect of IND in the small intestine, although the rate of caspase-3 activation was only moderate, possibly due to the mild to moderate severity of enteropathy induced by the present experimental protocol. We also found that IND-induced apoptosis was inversely modulated by LPAR2 activity, suggesting that increased sensitivity and resistance to IND in *Lpar2*^*−/−*^ and DBIBB-treated mice, respectively, is in part caused by the well-established antiapoptotic properties of this receptor [24–26]. The inhibitory effect of DBIBB treatment on intestinal apoptosis, however, was much less pronounced than in a previous study using mice exposed to total- and partial-body irradiation [25]. Although this difference can certainly be caused by obvious differences between the two models in terms of disease severity and treatment duration, we hypothesized that it may also be related to the different immune status of animals. Irradiation of mice compromises severely their immune system [66], which may mask the proinflammatory effects of DBIBB (as discussed below), and the potential ability of certain proapoptotic cytokines, such as interferon-γ and TNF-α [55] to counterbalance the direct antiapoptotic effect of LPAR2. Indeed, we found that the higher dose of DBIBB inhibited IND-induced apoptosis to a much greater extent in immunodeficient NOD/SCID mice than in normal mice, suggesting that impaired immunity tipped the balance between the direct antiapoptotic and indirect proapoptotic effects of DBIBB in favor of the former. On the other hand, it is noteworthy that the reduction in histological damage caused by DBIBB was comparable in normal and NOD/SCID mice, and the correlation between apoptosis and histological damage was weak in WT and *Lpar2*^*−/−*^ mice, which indicate that inhibition of epithelial apoptosis is likely to be one, but not the only factor that contributes to the protective effect of LPAR2 against IND-induced enteropathy.

Previous studies have shown that LPA stimulates COX-2 expression and prostaglandin E2 production in a human gastric cell line expressing LPAR2 [29, 30], whereas intestinal COX-2 expression is lower in *Lpar2*^*−/−*^ mice than in WT mice in a colitis-associated tumor model [34]. It is well-established that prostaglandins are key players in the maintenance of GI mucosal integrity [8, 9], and prostaglandins derived from not only COX-1 but also COX-2 contribute to mucosal defence against NSAID-induced GI injury [48, 49]. Although we did not assess directly the tissue levels of prostaglandins, we measured the expression of COX-2 as a marker of inflammation. We found that lack of LPAR2 resulted in lower intestinal COX-2 protein expression in both VEH- and IND-treated animals. In addition, although DBIBB per se had no effect on the expression of either COX-1 or COX-2 at the time of evaluation (i.e., one day after the second administration of this drug), it dose-dependently increased the enteropathy-induced elevation of COX-2 expression. Thus, intact LPAR2 signaling may contribute to maintenance of basal COX-2 expression in the intestine of mice, as also suggested by Lin et al. [34], and increased prostaglandin synthesis may be an additional mechanism that contributes to the mucosal protective effect of LPAR2 in NSAID enteropathy.

Although our results clearly demonstrate that LPAR2 contribute to the maintenance of mucosal integrity in NSAID enteropathy, they also provide evidence for the central role of LPAR2 in the regulation of inflammation associated with tissue damage. IND-induced intestinal injury triggered tissue inflammation within 24 h in WT mice, which was mainly characterized by the elevation of innate immunity-related inflammatory proteins and neutrophil recruitment, in agreement with previous reports [12, 44]. In *Lpar2*^*−/*−^ mice, however, the tissue levels of most inflammatory mediators measured at 24 h were much lower than in WT mice, with some of them being actually comparable to those of control (VEH-treated) animals, whereas activation of LPAR2 with DBIBB potentiated the IND-induced elevation of several cytokines and chemokines secreted by macrophages and other innate immune cells. Interestingly, combined treatment with IND and DBIBB also resulted in marked elevation of cytokines secreted mainly, although not exclusively, by T cells including IFN-γ, IL-2, IL-4, IL-5, IL-10, IL-13, and IL-17. Thus, LPAR2 does not only enhance mucosal defence against NSAIDs, but is also required for the development of a full-fledged inflammation in the NSAID-injured tissue. These results resonate well with previous observations that DSS-induced colitis is less severe in mice with deleted or silenced *Lpar2* gene [22, 34], LPAR2 receptor is expressed by and involved in the activation of numerous immune cells, including macrophages [22] and T cells [67, 68], and the ATX-LPA axis promotes the migration of macrophages [34] and CD3^+^ lymphocytes to the inflamed colonic mucosa [69].

In contrast to most inflammatory markers measured, MPO reached high levels already 6 h after IND treatment in *Lpar2*^*−/*−^ mice, and remained high and comparable to that of WT mice after 24 h. This result was in line with the histological finding that neutrophil accumulation started earlier in the mucosa of mice lacking LPAR2. In contrast, the number of neutrophils and expression of MPO were lower in mice treated with both DBIBB and IND, compared to those treated only with IND. These findings indicate that neutrophil activation closely paralleled the development of mucosal injury and suggest that neither inhibition nor activation of LPAR2 signaling had significant influence on the natural course of enteropathy-induced neutrophil recruitment. It is noteworthy that in previous studies LPAR1 rather than LPAR2 was shown to promote neutrophil migration in pneumonia [70] and production of the neutrophil chemoattractant IL-8 in human bronchial epithelial cells [71]. More importantly, reduced macrophage infiltration but unchanged neutrophil activation was reported in *Lpar2*^*−/−*^ mice with DSS colitis [34]. Undisturbed neutrophil recruitment in mice lacking LPAR2 may explain the apparently contradictory results that the intestines of *Lpar2*^*−/−*^ mice were shortened and showed moderate inflammatory alterations despite the low levels of most proinflammatory proteins, whereas the intestines of DBIBB-treated mice showed less histological damage, despite the higher levels of several cytokines and chemokines. Reactive oxygen species and proteolytic enzymes released by activated neutrophils have a pivotal role in the pathogenesis of NSAID-induced GI injury [10, 12], therefore maintained neutrophil recruitment in mice lacking LPAR2 could be sufficient to drive inflammation and amplify tissue damage, despite the lower activity of other immune cells.

Considering that LPAR2 may have a role in maintaining mucosal defence against NSAIDs, we also aimed to assess the intestinal expression of LPAR2, as well as the plasma activity of ATX, a major LPA-producer enzyme [19]. At present, little is known about the effects of NSAIDs on ATX activity and LPAR signaling. Chronic (one-month) treatment with low-dose aspirin was shown to reduce plasma LPA levels in patients with cerebrovascular disease [72], but acute ingestion of a higher dose of aspirin had no effect on plasma LPA levels or ATX activity in healthy volunteers [73]. In a further study, IND reduced ATX mRNA expression by 40% in cultured fibroblast-like synoviocytes of patients with rheumatoid arthritis, although the effect was not significant compared with the control [74]. Here we show that both plasma ATX activity and ATX protein expression were reduced by IND. In addition, IND decreased the small intestinal expression of both *Enpp2* and *Lpar2* in a time-dependent fashion. RNAscope analysis of the latter effect provided evidence for reduced *Lpar2* expression in both the epithelium and lamina propria, suggesting that IND-induced downregulation of *Lpar2* is a general effect and is not restricted to certain cell types.

Moreover, both inhibition of ATX activity and LPAR2 expression preceded the development of mucosal damage and inflammation, implying that downregulation of the ATX-LPA-LPAR2 axis may be an early event in the pathogenesis of IND-induced enteropathy. Whether these changes are hallmarks of NSAID enteropathy or only features of an early disease stage and/or mild intestinal inflammation will have to be examined in future experiments. It is plausible that more severe tissue inflammation at a later more advanced disease stage of enteropathy might overcome the direct effects of IND resulting in increased ATX and LPAR2 expression, similarly to the changes reported in inflammatory bowel disease and animal models of colitis [22, 33, 69, 75, 76].

The inhibitory effect of IND on ATX activity and LPAR2 expression also resonates with the current concept that increased activity of the ATX-LPA-LPAR2 axis promotes tumorigenesis [77], whereas NSAIDs, including IND, have chemopreventive properties [78–80]. Hence, it is reasonable to assume that downregulation of ATX and LPAR2 is an additional mechanism underlying the anticancer effect of NSAIDs, at least in certain cancer types in which LPAR2 is upregulated, such as colorectal cancer [31, 34].

A potential limitation of our study is that we used a single, large dose of IND to induce enteropathy. Although this protocol is widely used in animal studies [36, 37, 81, 82], it may not adequately represent the enteropathy caused by long-term NSAID use in humans. Nevertheless, the features of acute enteropathy caused by a large dose of IND in rodents, including focal intestinal ulcerations, increased tissue concentration of MPO, TNF-α, IL-1β and TLR4, and expansion of Gram-negative bacteria and loss of Gram-positives [37, 82, 83], are very similar to the changes caused by chronic administration of other NSAIDs, such as diclofenac [84, 85]. In addition, enteropathy caused by IND in rodents is similar in many aspects to that in humans, including the localization of ulcers, changes in intestinal permeability and responses to some treatments [5]. However, there are clearly differences between animal models and humans and our findings will have to be confirmed in human studies as well.

Finally, it should be noted that although our study focused on LPAR2, other types of LPARs may also have effect on NSAID enteropathy. LPAR1, for example, regulates intestinal mucosal repair [59] and epithelial barrier function in mice [86]. Likewise, LPAR5 is essential for the regeneration of intestinal epithelium [87] and is abundantly expressed by mouse small intestinal intraepithelial CD8 T cells [88], on which it acts as an inhibitory receptor [89]. Moreover, it has been shown recently that *Lpar5* is upregulated in enteroids isolated from *Lpar2*^*−/−*^ mice, and LPAR5 compensates for the loss of LPAR2 to maintain normal GI functions [90]. Further studies are needed to investigate the role of other LPARs and their interaction in the context of NSAID enteropathy.

**Fig. 11:**
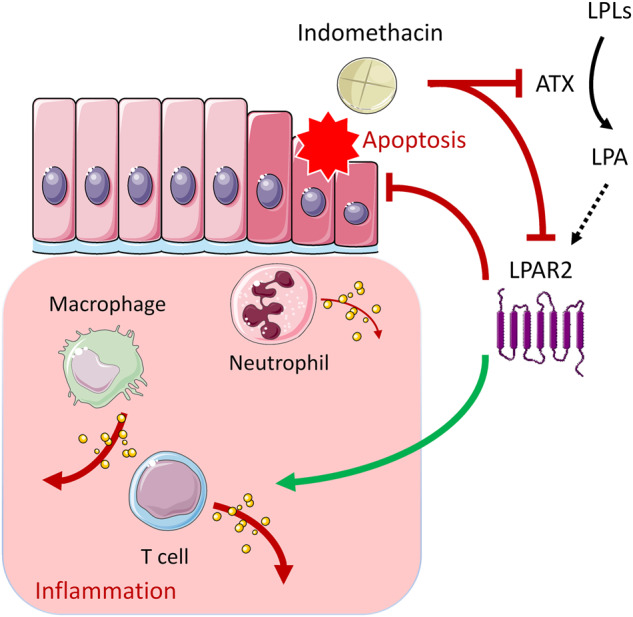
The proposed interaction between IND and LPAR2. IND treatment inhibits the plasma activity of autotaxin (ATX), and also downregulates the intestinal expression of LPAR2. LPAR2 exerts dual effect on IND-induced enteropathy; it mitigates the severity of mucosal damage, at least partly due to inhibition of intestinal apoptosis, but also potentiates the inflammatory reaction associated with ulceration. This latter effect involves the increased activation of several immune cells, including T cells, but not that of neutrophils, whose number and activity rather change in parallel with the severity of mucosal damage. LPLs: lysophospholipids.

In summary, our present study reveals the Janus face of LPAR2 signaling in NSAID enteropathy, which may limit the therapeutic applicability of LPAR2 ligands in this disease (Fig. 11). Here we showed for the first time that LPAR2 has a pivotal role in maintaining intestinal mucosal integrity against NSAIDs, because the lack of LPAR2 accelerates the development of NSAID enteropathy, whereas selective activation of LPAR2 reduces the severity of intestinal mucosal injury. Our study, however, also reveals the importance of LPAR2 in the development of tissue inflammation associated with enteropathy. LPAR2 activation, therefore, appears to be a double-edged sword in NSAID enteropathy, which protects the mucosa but at the same time, enhances the accompanying inflammatory reaction. This inflammation involves the increased activation of several immune cells, including T cells, but not that of neutrophils, whose numbers and activity change in parallel with the severity of mucosal damage. Our study also shows that IND can reduce the plasma activity of ATX and the intestinal expression of *Lpar2* mRNA earlier than the enteropathy develops, indicating that suppression of the ATX-LPA-LPAR2 axis may contribute to the initial topical damaging effect of IND to the mucosa.

## References

[1] Singh G (2000). Gastrointestinal complications of prescription and over-the-counter nonsteroidal anti-inflammatory drugs: a view from the ARAMIS database. Arthritis, Rheumatism, and Aging Medical Information System. Am J Ther.

[2] Lanas A, Scarpignato C (2006). Microbial flora in NSAID-induced intestinal damage: a role for antibiotics?. Digestion.

[3] Wallace JL (2013). Mechanisms, prevention and clinical implications of nonsteroidal anti-inflammatory drug-enteropathy. World J Gastroenterol.

[4] Boelsterli UA, Redinbo MR, Saitta KS (2013). Multiple NSAID-induced hits injure the small intestine: underlying mechanisms and novel strategies. Toxicol Sci.

[5] Bjarnason I, Scarpignato C, Holmgren E, Olszewski M, Rainsford KD, Lanas A (2018). Mechanisms of damage to the gastrointestinal tract from nonsteroidal anti-inflammatory drugs. Gastroenterology.

[6] Tomisato W, Tsutsumi S, Hoshino T, Hwang HJ, Mio M, Tsuchiya T (2004). Role of direct cytotoxic effects of NSAIDs in the induction of gastric lesions. Biochem Pharmacol.

[7] Tanaka K, Tomisato W, Hoshino T, Ishihara T, Namba T, Aburaya M (2005). Involvement of intracellular Ca^2+^ levels in nonsteroidal anti-inflammatory drug-induced apoptosis. J Biol Chem.

[8] Gyires K (2005). Gastric mucosal protection: from prostaglandins to gene-therapy. Curr Med Chem.

[9] Wallace JL (2008). Prostaglandins, NSAIDs, and gastric mucosal protection: why doesn’t the stomach digest itself?. Physiol Rev.

[10] Wallace J, Keenan C, Granger D (1990). Gastric ulceration induced by nonsteroidal anti inflammatory drugs is a neutrophil-dependent process. Am J Physiol.

[11] Konaka A, Kato S, Tanaka A, Kunikata T, Korolkiewicz R, Takeuchi K (1999). Roles of enterobacteria, nitric oxide and neutrophil in pathogenesis of indomethacin-induced small intestinal lesions in rats. Pharmacol Res.

[12] Beck PL, Xavier R, Lu N, Nanda NN, Dinauer M, Podolsky DK (2000). Mechanisms of NSAID-induced gastrointestinal injury defined using mutant mice. Gastroenterology.

[13] Watanabe T, Higuchi K, Kobata A, Nishio H, Tanigawa T, Shiba M (2008). Non-steroidal anti-inflammatory drug-induced small intestinal damage is Toll-like receptor 4 dependent. Gut.

[14] Wallace JL (2012). NSAID gastropathy and enteropathy: distinct pathogenesis likely necessitates distinct prevention strategies. Br J Pharmacol.

[15] Wallace JL, Syer S, Denou E, de Palma G, Vong L, McKnight W (2011). Proton pump inhibitors exacerbate NSAID-induced small intestinal injury by inducing dysbiosis. Gastroenterology.

[16] Taha AS, McCloskey C, McSkimming P, McConnachie A (2018). Misoprostol for small bowel ulcers in patients with obscure bleeding taking aspirin and non-steroidal anti-inflammatory drugs (MASTERS): a randomised, double-blind, placebo-controlled, phase 3 trial. Lancet Gastroenterol Hepatol.

[17] Yung YC, Stoddard NC, Chun J (2014). LPA receptor signaling: pharmacology, physiology, and pathophysiology. J Lipid Res.

[18] Geraldo LHM, Spohr T, Amaral RFD, Fonseca A, Garcia C, Mendes FA (2021). Role of lysophosphatidic acid and its receptors in health and disease: novel therapeutic strategies. Signal Transduct Target Ther.

[19] Aoki J, Inoue A, Okudaira S (2008). Two pathways for lysophosphatidic acid production. Biochim Biophys Acta.

[20] Tanaka T, Horiuchi G, Matsuoka M, Hirano K, Tokumura A, Koike T (2009). Formation of lysophosphatidic acid, a wound-healing lipid, during digestion of cabbage leaves. Biosci Biotechnol Biochem.

[21] Lin S, Yeruva S, He P, Singh AK, Zhang H, Chen M (2010). Lysophosphatidic acid stimulates the intestinal brush border Na^+^/H^+^ exchanger 3 and fluid absorption via LPA_5_ and NHERF2. Gastroenterology.

[22] Wang Z, Shi W, Tian D, Qin H, Vallance BA, Yang H (2020). Autotaxin stimulates LPA2 receptor in macrophages and exacerbates dextran sulfate sodium-induced acute colitis. J Mol Med (Berl).

[23] Deng W, Balazs L, Wang DA, Van Middlesworth L, Tigyi G, Johnson LR (2002). Lysophosphatidic acid protects and rescues intestinal epithelial cells from radiation- and chemotherapy-induced apoptosis. Gastroenterology.

[24] Deng W, Shuyu E, Tsukahara R, Valentine WJ, Durgam G, Gududuru V (2007). The lysophosphatidic acid type 2 receptor is required for protection against radiation-induced intestinal injury. Gastroenterology.

[25] Patil R, Szabo E, Fells JI, Balogh A, Lim KG, Fujiwara Y (2015). Combined mitigation of the gastrointestinal and hematopoietic acute radiation syndromes by an LPA2 receptor-specific nonlipid agonist. Chem Biol.

[26] Kuo B, Szabo E, Lee SC, Balogh A, Norman D, Inoue A (2018). The LPA_2_ receptor agonist Radioprotectin-1 spares Lgr5-positive intestinal stem cells from radiation injury in murine enteroids. Cell Signal.

[27] Shukla PK, Meena AS, Gangwar R, Szabo E, Balogh A, Chin Lee S (2020). LPAR2 receptor activation attenuates radiation-induced disruption of apical junctional complexes and mucosal barrier dysfunction in mouse colon. FASEB J.

[28] Tigyi GJ, Johnson LR, Lee SC, Norman DD, Szabo E, Balogh A (2019). Lysophosphatidic acid type 2 receptor agonists in targeted drug development offer broad therapeutic potential. J Lipid Res.

[29] Tanaka T, Ohmoto M, Morito K, Kondo H, Urikura M, Satouchi K (2014). Type 2 lysophosphatidic acid receptor in gastric surface mucous cells: possible implication of prostaglandin E2 production. Biofactors.

[30] Afroz S, Yagi A, Fujikawa K, Rahman MM, Morito K, Fukuta T (2018). Lysophosphatidic acid in medicinal herbs enhances prostaglandin E_2_ and protects against indomethacin-induced gastric cell damage in vivo and in vitro. Prostaglandins Other Lipid Mediat.

[31] Yun CC, Sun H, Wang D, Rusovici R, Castleberry A, Hall RA (2005). LPA2 receptor mediates mitogenic signals in human colon cancer cells. Am J Physiol Cell Physiol.

[32] Yamashita H, Kitayama J, Shida D, Ishikawa M, Hama K, Aoki J (2006). Differential expression of lysophosphatidic acid receptor-2 in intestinal and diffuse type gastric cancer. J Surg Oncol.

[33] Dong YL, Duan XY, Liu YJ, Fan H, Xu M, Chen QY (2019). Autotaxin-lysophosphatidic acid axis blockade improves inflammation by regulating Th17 cell differentiation in DSS-induced chronic colitis mice. Inflammation.

[34] Lin S, Wang D, Iyer S, Ghaleb AM, Shim H, Yang VW (2009). The absence of LPA2 attenuates tumor formation in an experimental model of colitis-associated cancer. Gastroenterology.

[35] Contos JJ, Ishii I, Fukushima N, Kingsbury MA, Ye X, Kawamura S (2002). Characterization of lpa_2_ (Edg4) and lpa_1_/lpa_2_ (Edg2/Edg4) lysophosphatidic acid receptor knockout mice: signaling deficits without obvious phenotypic abnormality attributable to lpa_2_. Mol Cell Biol.

[36] Liang X, Bittinger K, Li X, Abernethy DR, Bushman FD, FitzGerald GA (2015). Bidirectional interactions between indomethacin and the murine intestinal microbiota. Elife.

[37] Lazar B, Laszlo SB, Hutka B, Toth AS, Mohammadzadeh A, Berekmeri E (2021). A comprehensive time course and correlation analysis of indomethacin-induced inflammation, bile acid alterations and dysbiosis in the rat small intestine. Biochem Pharmacol.

[38] Knowlden SA, Hillman SE, Chapman TJ, Patil R, Miller DD, Tigyi G (2016). Novel inhibitory effect of a lysophosphatidic acid 2 agonist on allergen-driven airway inflammation. Am J Respir Cell Mol Biol.

[39] Laszlo SB, Lazar B, Brenner GB, Makkos A, Balogh M, Al-Khrasani M (2020). Chronic treatment with rofecoxib but not ischemic preconditioning of the myocardium ameliorates early intestinal damage following cardiac ischemia/reperfusion injury in rats. Biochem Pharmacol.

[40] Zadori ZS, Toth VE, Feher A, Al-Khrasani M, Puskar Z, Kozsurek M (2016). Inhibition of alpha2A-adrenoceptors ameliorates dextran sulfate sodium-induced acute intestinal inflammation in mice. J Pharmacol Exp Ther.

[41] Bankhead P, Loughrey MB, Fernandez JA, Dombrowski Y, McArt DG, Dunne PD (2017). QuPath: open source software for digital pathology image analysis. Sci Rep.

[42] Yoshimi T, Yamagishi Y, Kanegawa I, Suda M, Saiki R, Tanaka KI, et al. Study of the inhibitory effects of enteral nutrition formula on indomethacin-induced gastric lesions in mice. Nutrients. 2019;11:3058. 10.3390/nu11123058 PMC6949949 31847337

[43] Banerjee S, Norman DD, Lee SC, Parrill AL, Pham TC, Baker DL (2017). Highly potent non-carboxylic acid autotaxin inhibitors reduce melanoma metastasis and chemotherapeutic resistance of breast cancer stem cells. J Med Chem.

[44] Koga H, Aoyagi K, Matsumoto T, Iida M, Fujishima M (1999). Experimental enteropathy in athymic and euthymic rats: synergistic role of lipopolysaccharide and indomethacin. Am J Physiol.

[45] Stadnyk A, Dollard C, Issekutz T, Issekutz A (2002). Neutrophil migration into indomethacin induced rat small intestinal injury is CD11a/CD18 and CD11b/CD18 co-dependent. Gut.

[46] Bertrand V, Guimbaud R, Tulliez M, Mauprivez C, Sogni P, Couturier D (1998). Increase in tumor necrosis factor-alpha production linked to the toxicity of indomethacin for the rat small intestine. Br J Pharmacol.

[47] Tsatsanis C, Androulidaki A, Venihaki M, Margioris AN (2006). Signalling networks regulating cyclooxygenase-2. Int J Biochem Cell Biol.

[48] Wallace JL, McKnight W, Reuter BK, Vergnolle N (2000). NSAID-induced gastric damage in rats: requirement for inhibition of both cyclooxygenase 1 and 2. Gastroenterology.

[49] Tanaka A, Hase S, Miyazawa T, Ohno R, Takeuchi K (2002). Role of cyclooxygenase (COX)-1 and COX-2 inhibition in nonsteroidal anti-inflammatory drug-induced intestinal damage in rats: relation to various pathogenic events. J Pharmacol Exp Ther.

[50] Doni A, Stravalaci M, Inforzato A, Magrini E, Mantovani A, Garlanda C (2019). The long pentraxin PTX3 as a link between innate immunity, tissue remodeling, and cancer. Front Immunol.

[51] Chow JY, Li ZJ, Wu WK, Cho CH (2013). Cathelicidin a potential therapeutic peptide for gastrointestinal inflammation and cancer. World J Gastroenterol.

[52] Lin S, Lee SJ, Shim H, Chun J, Yun CC (2010). The absence of LPA receptor 2 reduces the tumorigenesis by ApcMin mutation in the intestine. Am J Physiol Gastrointest Liver Physiol.

[53] Maity P, Bindu S, Dey S, Goyal M, Alam A, Pal C (2009). Indomethacin, a non-steroidal anti-inflammatory drug, develops gastropathy by inducing reactive oxygen species-mediated mitochondrial pathology and associated apoptosis in gastric mucosa: a novel role of mitochondrial aconitase oxidation. J Biol Chem.

[54] Fukumoto K, Naito Y, Takagi T, Yamada S, Horie R, Inoue K (2011). Role of tumor necrosis factor-alpha in the pathogenesis of indomethacin-induced small intestinal injury in mice. Int J Mol Med.

[55] Andrews C, McLean MH, Durum SK (2018). Cytokine tuning of intestinal epithelial function. Front Immunol.

[56] Blander JM (2016). Death in the intestinal epithelium-basic biology and implications for inflammatory bowel disease. FEBS J.

[57] Patton J, Bonne-Année S, Deckman J, Hess J, Torigian A, Nolan T (2018). Methylprednisolone acetate induces, and Δ7-dafachronic acid suppresses, *Strongyloides stercoralis* hyperinfection in NSG mice. Proc Natl Acad Sci USA.

[58] Sturm A, Sudermann T, Schulte K, Goebell H, Dignass A (1999). Modulation of intestinal epithelial wound healing in vitro and in vivo by lysophosphatidic acid. Gastroenterology.

[59] Lee SJ, Leoni G, Neumann PA, Chun J, Nusrat A, Yun CC (2013). Distinct phospholipase C-beta isozymes mediate lysophosphatidic acid receptor 1 effects on intestinal epithelial homeostasis and wound closure. Mol Cell Biol.

[60] Adachi M, Horiuchi G, Ikematsu N, Tanaka T, Terao J, Satouchi K (2011). Intragastrically administered lysophosphatidic acids protect against gastric ulcer in rats under water-immersion restraint stress. Dig Dis Sci.

[61] Tanaka T, Morito K, Kinoshita M, Ohmoto M, Urikura M, Satouchi K (2013). Orally administered phosphatidic acids and lysophosphatidic acids ameliorate aspirin-induced stomach mucosal injury in mice. Dig Dis Sci.

[62] Thompson KE, Ray RM, Alli S, Ge W, Boler A, Shannon McCool W (2018). Prevention and treatment of secretory diarrhea by the lysophosphatidic acid analog Rx100. Exp Biol Med (Maywood).

[63] Tsutsumi S, Gotoh T, Tomisato W, Mima S, Hoshino T, Hwang HJ (2004). Endoplasmic reticulum stress response is involved in nonsteroidal anti-inflammatory drug-induced apoptosis. Cell Death Differ.

[64] Omatsu T, Naito Y, Handa O, Mizushima K, Hayashi N, Qin Y (2010). Reactive oxygen species-quenching and anti-apoptotic effect of polaprezinc on indomethacin-induced small intestinal epithelial cell injury. J Gastroenterol.

[65] Kumar S (2007). Caspase function in programmed cell death. Cell Death Differ.

[66] Booth C, Tudor G, Tudor J, Katz BP, MacVittie TJ (2012). Acute gastrointestinal syndrome in high-dose irradiated mice. Health Phys.

[67] Zheng Y, Kong Y, Goetzl EJ (2001). Lysophosphatidic acid receptor-selective effects on Jurkat T cell migration through a Matrigel model basement membrane. J Immunol.

[68] Knowlden S, Georas SN (2014). The autotaxin-LPA axis emerges as a novel regulator of lymphocyte homing and inflammation. J Immunol.

[69] Hozumi H, Hokari R, Kurihara C, Narimatsu K, Sato H, Sato S (2013). Involvement of autotaxin/lysophospholipase D expression in intestinal vessels in aggravation of intestinal damage through lymphocyte migration. Lab Invest.

[70] Rahaman M, Costello RW, Belmonte KE, Gendy SS, Walsh MT (2006). Neutrophil sphingosine 1-phosphate and lysophosphatidic acid receptors in pneumonia. Am J Respir Cell Mol Biol.

[71] Saatian B, Zhao Y, He D, Georas SN, Watkins T, Spannhake EW (2006). Transcriptional regulation of lysophosphatidic acid-induced interleukin-8 expression and secretion by p38 MAPK and JNK in human bronchial epithelial cells. Biochem J.

[72] Li Z, Yu Z, Wang D, Ju W, Zhan X, Wu Q (2008). Influence of acetylsalicylate on plasma lysophosphatidic acid level in patients with ischemic cerebral vascular diseases. Neurol Res.

[73] Block RC, Duff R, Lawrence P, Kakinami L, Brenna JT, Shearer GC (2010). The effects of EPA, DHA, and aspirin ingestion on plasma lysophospholipids and autotaxin. Prostaglandins Leukot Ess Fat Acids.

[74] Kehlen A, Lauterbach R, Santos A, Thiele K, Kabisch U, Weber E (2001). IL-1 beta- and IL-4-induced down-regulation of autotaxin mRNA and PC-1 in fibroblast-like synoviocytes of patients with rheumatoid arthritis (RA). Clin Exp Immunol.

[75] Zhang P, Chen Y, Zhang T, Zhu J, Zhao L, Li J (2018). Deficiency of alkaline SMase enhances dextran sulfate sodium-induced colitis in mice with upregulation of autotaxin. J Lipid Res.

[76] Lin S, Haque A, Raeman R, Guo L, He P, Denning TL (2019). Autotaxin determines colitis severity in mice and is secreted by B cells in the colon. FASEB J.

[77] Mills GB, Moolenaar WH (2003). The emerging role of lysophosphatidic acid in cancer. Nat Rev Cancer.

[78] Thun M, Henley S, Patrono C (2002). Nonsteroidal anti-inflammatory drugs as anticancer agents: mechanistic, pharmacologic, and clinical issues. J Natl Cancer Inst.

[79] Hull MA, Gardner SH, Hawcroft G (2003). Activity of the non-steroidal anti-inflammatory drug indomethacin against colorectal cancer. Cancer Treat Rev.

[80] Wong RSY (2019). Role of nonsteroidal anti-inflammatory drugs (NSAIDs) in cancer prevention and cancer promotion. Adv Pharmacol Sci.

[81] Kent TH, Cardelli RM, Stamler FW (1969). Small intestinal ulcers and intestinal flora in rats given indomethacin. Am J Pathol.

[82] Watanabe T, Nishio H, Tanigawa T, Yamagami H, Okazaki H, Watanabe K (2009). Probiotic Lactobacillus casei strain Shirota prevents indomethacin-induced small intestinal injury: involvement of lactic acid. Am J Physiol Gastrointest Liver Physiol.

[83] Zadori ZS, Kiraly K, Al-Khrasani M, Gyires K (2023). Interactions between NSAIDs, opioids and the gut microbiota—future perspectives in the management of inflammation and pain. Pharmacol Ther.

[84] Colucci R, Pellegrini C, Fornai M, Tirotta E, Antonioli L, Renzulli C (2018). Pathophysiology of NSAID-associated intestinal lesions in the rat: luminal bacteria and mucosal inflammation as targets for prevention. Front Pharmacol.

[85] D’Antongiovanni V, Antonioli L, Benvenuti L, Pellegrini C, Di Salvo C, Calvigioni M, et al. Use of Saccharomyces boulardii CNCM I-745 as therapeutic strategy for prevention of nonsteroidal anti-inflammatory drug-induced intestinal injury. Br J Pharmacol. 2023; in press. 10.1111/bph.16200 37519261

[86] Lin S, Han Y, Jenkin K, Lee SJ, Sasaki M, Klapproth JM (2018). Lysophosphatidic acid receptor 1 is important for intestinal epithelial barrier function and susceptibility to colitis. Am J Pathol.

[87] Liang Z, He P, Han Y, Yun CC (2022). Survival of stem cells and progenitors in the intestine is regulated by LPA_5_-dependent signaling. Cell Mol Gastroenterol Hepatol.

[88] Kotarsky K, Boketoft A, Bristulf J, Nilsson NE, Norberg A, Hansson S (2006). Lysophosphatidic acid binds to and activates GPR92, a G protein-coupled receptor highly expressed in gastrointestinal lymphocytes. J Pharmacol Exp Ther.

[89] Oda SK, Strauch P, Fujiwara Y, Al-Shami A, Oravecz T, Tigyi G (2013). Lysophosphatidic acid inhibits CD8 T cell activation and control of tumor progression. Cancer Immunol Res.

[90] Liang Z, Yun CC. Compensatory upregulation of LPA_2_ and activation of the PI3K-Akt pathway prevent LPA_5_-dependent loss of intestinal epithelial cells in intestinal organoids. Cells. 2022;11:2243. 10.3390/cells11142243 PMC9324510 35883686

